# Essentials of Vaccination

**Published:** 1886-10

**Authors:** 


Essentials of Vaccination: *a compilation of facts relating to Vaccine Inoculation and its Influence in the Prevention of Small Pox. By* W. A. Hardaway, M. D., *12mo, pp. 145. St. Louis:* J. H. Chambers & Co., 1886. *Chicago:* W. T. Keener.

The author has succeeded in presenting the most essential facts of vaccination, in a limited compass, and in an agreeable manner. The necessity for this book is apparent in the erroneous views held by many of the profession regarding vaccination, and the careless manner in which the operation is too often performed.

Great stress is laid on the efficiency of vaccination in estimating the protection afforded. Marston's tables are quoted, in which he clearly proves that the best vaccination is some thirty times more protective than the poorest. The author clearly gives his preference for bovine over humanized virus, in which it is probable that the majority of the profession in this country will agree with him. Excellent instruction is given in the methods of obtaining and storing virus, and also the manner in which the operation should be performed. Here the work seems to be open to criticism. He advises the use of a *clean* lancet, or else the ivory point, in scarifying, which is recommended rather than puncture or incision. Nothing is said about the arm being clean, also the hands of the operator, and using Lister's protective, and a layer of antiseptic cotton confined by a wire vaccination protector; in other words, carefully protecting the wound from all external infection. The adoption of this precaution will, we are sure, exclude many of the untoward results mentioned in the book. The work closes with a concise account of the principal objections to vaccination.

